# Experience of physical violence and mental health among young men and women: a population-based study in Sweden

**DOI:** 10.1186/1471-2458-14-29

**Published:** 2014-01-11

**Authors:** Maria Fridh, Martin Lindström, Maria Rosvall

**Affiliations:** 1 Department of Clinical Sciences, Social Medicine and Health Policy, CRC, Jan Waldenströms gata 35, Scania University Hospital, Lund University, SE-205 02 Malmö, Sweden; 2 Centre for Economic Demography, Lund University School of Economics and Management, SE-220 07 Lund, Sweden

**Keywords:** Physical violence, Psychological health, Trust, Epidemiology, Sweden

## Abstract

**Background:**

In Sweden mental ill-health has increased among the young, especially among young women. Our aim was to investigate the association between experience of physical violence during the past year and self rated psychological health among young men and women.

**Methods:**

The study population consisted of men (n = 2,624) and women (n = 3,569) aged 18–34 years who participated in the 2008 public health survey study in Skåne. The survey was a cross-sectional stratified random sample postal questionnaire study with a 54.1% participation rate. Associations were investigated by logistic regression models.

**Results:**

The prevalence of poor psychological health was 18.9% among men and 27.7% among women. One in ten men and one in twenty women had experienced physical violence during the past year. Most men were violated in public places, while women were most often violated at home. Women who had experienced violence during the past year showed more than doubled odds of poor psychological health, odds ratio (OR): 2.66 (95% confidence interval (CI): 2.00, 3.53). Such an association could not be seen in men OR: 1.12 (95% CI: 0.85, 1.47). Adjustment for covariates (i.e. age, country of birth, socioeconomic status, economic stress, alcohol risk consumption, emotional support, instrumental support and generalized trust in other people) did not change the association found among women.

**Conclusion:**

Violated women, but not men, showed nearly doubled odds of poor psychological health after multiple adjustments. There was also a gender difference regarding location of violence. Awareness of gender differences regarding context and mental impact of violence may assist public health workers in reducing the consequences of violence and to design preventive strategies.

## Background

Exposure to violence is a public health issue with long term human and economic costs [1]. In the Swedish society there has been a marked increase of violence since the beginning of the 1980s according to crime statistics [1]. The risk of violence is highest among young persons (16–24 years), and higher among men than women [1]. Most violence experienced by men is perpetrated by other men [1,2], primarily takes place in public spaces [1,3,4] and the perpetrator of a man is often unknown to the victim [1,2,5,6]. Men are more often hospitalized due to assault injuries and more often die as a result of violence than women [1], but four to five times as many women as men die as a result of partner violence [1]. Most violence experienced by women is perpetrated by men, primarily occurs at home [1,3] and the perpetrator is usually known to the woman [1,2,5,7]. Women abused by a partner are often exposed to repeated violence [8] and domestic violence has been shown to have serious consequences on physical and mental health, both in a short and long perspective [1,9]. Primarily due to costs of psychiatric treatment, male and female victims of violence have higher total healthcare costs than men and women not exposed to violence [10]. Still, many victims of violence have reported that they would have needed more health care [7,11]. Studies have found associations between exposure to violence and social factors such as economic stress [11], ethnicity [3], socioeconomic status [3,12], as well as psychosocial factors such as social support [3,13,14] and trust [3]. Furthermore, several studies have shown an association between alcohol risk consumption and exposure to violence [3,6,10,15].

In Sweden poor self reported psychological health is most prevalent among young women. The prevalence of poor self reported psychological health has increased over the last decades in surveys, reflected by an increase in the incidence of depression, anxiety and self-harm among young men and women in psychiatric hospital care statistics [16]. Poor psychological health has been shown to be associated with economic stress [17,18], ethnicity [17,18], socioeconomic status [17], emotional and instrumental support [18], trust [17-19] and alcohol risk consumption [20,21]. There are some earlier population-based studies on adults’ experiences of physical violence and mental health measured as self reported psychological distress [2,6,7,11-14,22-24]. Five of these were conducted on both men and women [2,6,11,22,23] and five on women only [7,12-14,24]. Three were conducted on students [2,6,23] and two researched a younger population up to 45 years of age [11,22]. Three of these studies used the General Health Questionnaire 12 (GHQ-12) to evaluate psychological distress [6,14,23], while the others used a range of different instruments. GHQ-12 has the advantage of being an internationally well validated measure of psychological ill-health in the general population [25,26]. To the best of our knowledge, there is no earlier study that has investigated the associations between experience of physical violence and self rated psychological health measured by GHQ-12 in relation to social factors (socioeconomic status, ethnicity, economic stress) and psychosocial factors (emotional and instrumental support, trust) and alcohol use, in the same study.

The aim of this study was to investigate the association between experience of physical violence during the past year and self rated psychological health in relation to the above-mentioned factors in both men and women. Furthermore, the present study was to explore the setting of violence.

## Methods

### Study population

The 2008 public health survey in Skåne in southern Sweden was a cross-sectional stratified random sample study. The primary purpose of this public health survey was to map out the health situation in the general population of Scania, Sweden, in the year 2008 [27]. A total of 28,198 persons aged 18–80 years answered the postal questionnaire, representing 54.1% of the net selection [27]. The present research study is a secondary study based on a subpopulation of those included in the public health survey, i.e. participants in the age interval 18–34 years, which included a total of 6,193 respondents (2,624 men and 3,569 women). The data from the stratified random sample study was weighted by various factors, e.g., age, sex and administration area through a weighting variable [27]. The differences between unweighted and weighted data were very small (data not shown). Ethical approval to conduct the research study was granted by the Ethical Committee at Lund University, Sweden (No. 2010/343).

### Definitions

#### Dependent variable

*Self reported psychological health (GHQ-12)* included twelve items reflecting different aspects of psychological health, such as anxiety and depression, the ability to perform daily activities and the ability to cope with everyday problems during a time period of the last few weeks. Each item had four response categories, e.g. “Better than usual”, “Same as usual”. “Less than usual” and “Much less than usual”. Scoring was according to the GHQ method (0,0,1,1) instead of the Likert method (0,1,2,3) [28]. The answers to the 12 items were dichotomized into “good” or “poor” psychological health. If three or more of the twelve items denoted “poor” psychological health, the respondent’s general psychological health (GHQ-12) was defined as “poor”. This cut-off has been widely used for many years in Sweden and in many studies abroad [27,29,30]. The GHQ12- instrument is the shortest (other GHQ measures contain for instance 28 or 60 items), but has been shown to be a very robust measure of psychological health [25].

#### Independent variables

The *age* interval 18–34 years was analyzed in this study. Age adjustments in tables were conducted with age as a continuous variable within the age interval 18–34 years.

*Born in Sweden/born in other Scandinavian countries/born in the rest of Europe/born outside Europe.* The participants were categorized according to place of birth.

*Socioeconomic status (SES)* by occupation included the employed categories higher non-manual employees, medium level non-manual employees, low level non-manual employees, skilled manual workers and unskilled manual workers as well as self-employed/farmers. The groups outside the workforce comprised the unemployed, the early retired (for health or early retirement entitlement in the employment contract reasons), students, and persons on long term sick leave. Furthermore, there was the group unclassified.

*Alcohol risk consumption* was estimated by an index of three questions: how often you drink alcohol, how much alcohol you typically drink and how often you drink a large amount on one occasion. The index can take a point value between 0 and 12. Alcohol risk consumption was defined as 8–12 points for men and 6–12 points for women. Additionally, those who had been intoxicated 2–3 times a month or more often were defined as alcohol risk consumers. These questions have been used by the National Public Health Reports of Sweden to define alcohol risk consumption [31].

*Emotional support* was assessed with the question “Do you feel that you have one or several persons who can give you sufficient personal support to handle the stress and problems of life?”. The four alternative answers were: “Yes, I am absolutely certain to get such support”, “Yes, possibly”, “Not certain”, and “No”. The item was dichotomized, and the three latter alternatives were classified as low emotional support.

*Instrumental support* was assessed with the question “Can you get help from one or several persons in case of illness or practical problems (to borrow things, repair things, write a letter, get advice or information)”? This item had similar alternative answers as emotional support and was dichotomized correspondingly.

*Economic stress* was assessed with the item “How often during the past twelve months have you had problems paying your bills?” with the four alternative answers: “Never”, “Occasionally”, “Every second month” and “Every month”.

*Generalized (horizontal) trust in other people* was appraised by the item “Generally, you can trust other people” with the four alternative answers: “Do not agree at all”, “Do not agree”, “Agree”, and “Completely agree”. These alternatives were dichotomized with the two first alternatives indicating low trust and the two latter high trust. This item has been used with four optional answers in most previous investigations collapsing the alternatives in the same way [17,32].

*Experience of physical violence* during the past year was assessed with the question: “Have you at any time during the past twelve months been exposed to physical violence?” with the alternatives “Yes” and “No”.

*Location of physical violence* during the past year was assessed with the supplementary question: “If yes, where did this occur? You may tick several options” with the alternative answers: “At work/at school”, “At home”, “In somebody else’s home/in the neighborhood”, “In a public place/at a venue/on a train, bus, subway” and “Somewhere else”.

#### Statistics

The prevalence (%) of poor self rated psychological health, age, country of birth, socioeconomic status, economic stress, alcohol risk consumption, emotional support, instrumental support and trust were stratified by sex in the two groups who had, alternatively had not, experienced physical violence during the past year (Table 1). The odds ratios with 95% confidence intervals (OR:s, 95% CI) of poor self rated psychological health were calculated in a bivariate model stratified by sex and according to age, country of birth, socioeconomic status, economic stress, alcohol risk consumption, emotional support, instrumental support, horizontal trust and experience of physical violence during the past year using logistic regression modeling (Table 2). Age-adjusted and multiple adjusted odds ratios and 95% confidence intervals of poor self rated psychological health according to experience of physical violence during the past year were calculated for men and women using logistic regression modeling (Table 3). Adjustments were made for age, country of origin, socioeconomic status, economic stress, alcohol risk drinking, emotional support, instrumental support and trust. The statistical analyses were performed using IBM SPSS Statistics version 19.

**Table 1:** Characteristics (%) of men and women exposed (yes) and unexposed (no) to physical violence during the past year

	**Men**		**Women**	
	**Physical violence**		**Physical violence**	
	**Yes**	**No**	**Yes**	**No**
**Psychological health**				
Good	79.3	81.2	49.5	73.5
Poor	20.7	18.8	50.5	26.5
**Age**				
18-24	68.7	38.8	54.5	40.3
25-34	31.3	61.2	45.5	59.7
**Country of origin**				
Sweden	89.8	81.5	82.1	81.4
Other Nordic countries	1.1	2.4	0.9	2.4
The rest of Europe	2.7	9.1	7.5	8.1
Outside Europe	6.5	7.0	9.4	8.1
**Socioeconomic status**				
Higher non-manual	4.5	9.0	1.4	7.3
Medium non-manual	2.5	12.0	9.1	15.1
Lower non-manual	4.5	4.7	8.7	8.4
Skilled manual	12.9	11.4	13.9	8.5
Unskilled manual	21.8	15.2	22.1	14.5
Self-employed/farmer	2.2	4.0	0.5	2.3
Early retired	0.3	0.4	1.4	0.5
Unemployed	10.4	6.0	11.1	6.4
Student	23.8	23.0	22.1	26.6
Unclassified	16.8	13.6	8.7	9.4
Long term sick leave	0.3	0.6	1.0	1.0
**Economic stress**				
Never	64.2	74.1	48.1	65.2
Occasionally	26.8	18.1	28.1	24.7
Half the year	3.4	4.4	14.3	5.4
Every month	5.6	3.4	9.5	4.8
**Alcohol risk consumption**				
No	46.5	71.1	59.0	79.6
Yes	53.5	28.9	41.0	20.4
**Emotional support**				
High	62.0	68.2	48.8	75.2
Low	38.0	31.8	51.2	24.8
**Instrumental support**				
High	73.9	79.5	68.2	80.4
Low	26.1	20.5	31.8	19.6
**Trust (horizontal)**				
High	42.7	60.5	37.2	56.1
Low	57.3	39.5	62.8	43.9

Prevalences (%) of psychological health, age, country of origin, socioeconomic status, economic stress, alcohol risk consumption, emotional support, instrumental support and trust among men and women who had/had not experienced physical violence during the past year. Men (n = 2,624), women (n = 3,569), and total (n = 6,193) aged 18–34 years. The public health survey in Skåne 2008.

**Table 2:** Bivariate analyses of sociodemographic factors, psychosocial factors, alcohol risk consumption, and exposure to physical violence in relation to poor self rated psychological health

	**Men**		**Women**	
	**%**	**OR (95% CI)**	**%**	**OR (95% CI)**
**Age**				
18-24	19.6	1.00	31.9	1.00
25-34	18.3	0.92 (0.78–1.09)	24.9	0.70 (0.61–0.81)
**Country of origin**				
Sweden	18.1	1.00	27.2	1.00
Other Nordic countries	16.3	0.85 (0.47–1.53)	25.8	0.93 (0.59–1.47)
The rest of Europe	20.9	1.20 (0.91–1.60)	30.8	1.19 (0.93–1.52)
Outside Europe	26.3	1.62 (1.21–2.17)	30.9	1.20 (0.94–1.53)
**Socioeconomic status**				
Higher non–manual	20.3	1.00	23.2	1.00
Medium non–manual	14.7	0.68 (0.46–1.00)	22.9	0.98 (0.70–1.38)
Lower non–manual	15.4	0.72 (0.44–1.18)	26.9	1.21 (0.84–1.74)
Skilled manual	12.1	0.54 (0.36–0.80)	22.6	0.96 (0.66–1.39)
Unskilled manual	14.2	0.65 (0.46–0.93)	27.2	1.24 (0.89–1.71)
Self–employed/farmer	13.5	0.61 (0.35–1.07)	27.2	1.23 (0.72–2.10)
Early retired	16.7	0.85 (0.20–3.69)	55.0	4.14 (1.66–10.37)
Unemployed	37.0	2.30 (1.56–3.38)	45.6	2.76 (1.92–3.97)
Student	21.4	1.06 (0.77–1.47)	28.7	1.33 (0.98–1.80)
Unclassified	21.1	1.05 (0.74–1.48)	23.5	1.02 (0.71–1.46)
Long term sick leave	45.5	3.11 (1.27–7.61)	74.4	9.28 (4.35–19.82)
**Economic stress**				
Never	16.4	1.00	23.2	1.00
Occasionally	19.2	1.22 (0.98–1.51)	31.2	1.50 (1.28–1.77)
Half the year	38.4	3.19 (2.28–4.45)	43.8	2.60 (1.98–3.42)
Every month	42.7	3.80 (2.65–5.45)	49.5	3.28 (2.46–4.38)
**Alcohol risk consumption**				
No	19.5	1.00	25.8	1.00
Yes	17.5	0.87 (0.73–1.04)	34.1	1.49 (1.27–1.75)
**Emotional support**				
High	13.9	1.00	21.5	1.00
Low	29.2	2.55 (2.16–3.02)	45.4	3.03 (2.61–3.51)
**Instrumental support**				
High	16.2	1.00	23.4	1.00
Low	29.0	2.10 (1.75–2.53)	45.0	2.68 (2.29–3.15)
**Trust (horizontal)**				
High	15.0	1.00	21.1	1.00
Low	24.1	1.80 (1.52–2.12)	35.6	2.08 (1.81–2.39)
**Experience of physical violence during the past year**				
No	18.8	1.00	26.5	1.00
Yes	20.7	1.13 (0.86–1.47)	50.5	2.81 (2.12–3.72)

Prevalences (%) and odds ratios (OR, 95% CI) in bivariate analyses of poor self rated psychological health according to age, country of origin, socioeconomic status, economic stress, alcohol risk consumption, emotional support, instrumental support, trust and experience of physical violence during the past year. Men (n = 2,624) and women (n = 3,569) aged 18–34 years. The public health survey in Skåne 2008.

**Table 3:** Associations of exposure to physical violence and poor psychological health in multiple adjusted analyses

**Men**				
Violence	**OR (95% CI)**^**a**^	**OR (95% CI)**^**b**^	**OR (95% CI)**^**c**^	**OR (95% CI)**^**d**^
No	1.00	1.00	1.00	1.00
Yes	1.12 (0.85–1.47)	1.14 (0.86–1.49)	1.20 (0.91–1.58)	1.02 (0.76–1.36)
	**OR (95% CI)**^**e**^	**OR (95% CI)**^**f**^	**OR (95% CI)**^**g**^	**OR (95% CI)**^**h**^
No	1.00	1.00	1.00	1.00
Yes	1.06 (0.79–1.42)	1.00 (0.74–1.35)	1.00 (0.74–1.34)	1.00 (0.72–1.31)
**Women**				
Violence	**OR (95% CI)**^**a**^	**OR (95% CI)**^**b**^	**OR (95% CI)**^**c**^	**OR (95% CI)**^**d**^
No	1.00	1.00	1.00	1.00
Yes	2.66 (2.00–3.53)	2.66 (2.00–3.54)	2.73 (2.05–3.63)	2.41 (1.79–3.23)
	**OR (95% CI)**^**e**^	**OR (95% CI)**^**f**^	**OR (95% CI)**^**g**^	**OR (95% CI)**^**h**^
No	1.00	1.00	1.00	1.00
Yes	2.29 (1.70–3.08)	1.85 (1.36–2.52)	1.83 (1.34–2.50)	1.70 (1.24–2.33)

^a^Adjusted for age.

^b^Adjusted for age and country of origin.

^c^Adjusted for age, country of origin, and socioeconomic status.

^d^Adjusted for age, country of origin, socioeconomic status and economic stress.

^e^Adjusted for age, country of origin, socioeconomic status, economic stress and alcohol risk consumption.

^f^Adjusted for age, country of origin, socioeconomic status, economic stress, alcohol risk consumption and emotional support.

^g^Adjusted for age, country of origin, socioeconomic status, economic stress, alcohol risk consumption, emotional support and instrumental support.

^h^Adjusted for age, country of origin, socioeconomic status, economic stress, alcohol risk consumption, emotional support, instrumental support and trust.

Age-adjusted and multiple adjusted odds ratios (OR, 95% CI) of poor self rated psychological health according to experience of physical violence during the past year. Men (n = 2,624) and women (n = 3,569) aged 18–34 years. The public health survey in Skåne 2008.

## Results

In this study 223 men and 174 women reported experience of physical violence during the past year, which corresponds to a prevalence of 9.7% for men and 5.0% for women (data not shown).

Table 1 displays the prevalences of different variables in 18–34 year old men and women who had, alternatively had not, experienced physical violence during the past year. Men who had been violated were often younger (18–24 years), were born in Sweden, were unskilled manual workers or unemployed, had economic stress, alcohol risk consumption, lower emotional and instrumental support and lower generalized trust in other people. A similar pattern was seen for women apart from country of origin. Women who had experienced physical violence during the past year reported poor psychological health at much higher levels, 50.5% compared to 26.5% among women not violated. Such a difference could not be seen among men for whom the corresponding figures were 20.7% and 18.8%, respectively.

**Figure 1:**
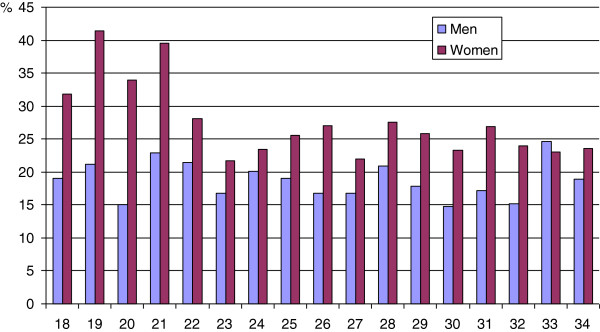
**Prevalence of poor psychological health (GHQ-12) by age.** Men (n= 2,624) and women (n= 3,569) aged 18-34 years. The public health survey in Skåne 2008.

Table 2 displays the prevalences and odds ratios of poor self rated psychological health in bivariate analyses. Women 18–24 years had poorer psychological health than women 25–34 years. Analysis by age showed that young women 18–21 years had the highest prevalence of poor psychological health with a peak of more than 40%, twice the rate of men the same age (Figure 1). Men born outside Europe had significantly higher odds of poor psychological health compared to men born in Sweden, while this pattern was not significant for women. Socioeconomic status showed a strong association with psychological health with generally higher odds of poor psychological health among those outside the workforce. For example, unemployed men and women showed more than doubled odds of poor psychological health compared to non-manual employees in higher positions. The odds of poor psychological health were higher among those with economic stress. Alcohol risk consumption was associated with poor psychological health among women OR = 1.49 (95% CI: 1.27, 1.75), but not among men OR = 0.87 (95% CI: 0.73, 1.04). Furthermore, psychosocial factors showed strong associations with poor psychological health among both men and women. The odds of poor psychological health were higher among those with low emotional support, low instrumental support and low trust. While women who had experienced violence during the past year showed more than doubled odds of poor psychological health, such a pattern could not be seen in men.

Table 3 shows the associations between exposure to physical violence and poor psychological health. The results showed significantly higher odds ratios of poor self rated psychological health among women with experience of physical violence during the past year compared to women unexposed to such violence throughout the age- and multiple adjusted logistic regression analyses. For example, in the age-adjusted model the odds ratio of poor self rated psychological health among women with experience of physical violence compared to women with no such experience was 2.66 (95% CI: 2.00, 3.53). Identical analyses showed no such associations in men with an age-adjusted OR of 1.12 (95% CI: 0.85, 1.47).

**Figure 2:**
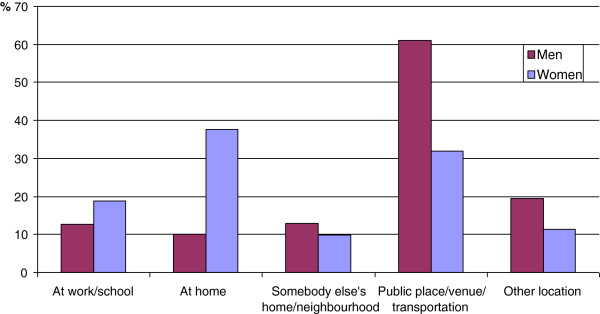
**The setting of exposure to physical violence.** Men (n= 223) and women (n= 174) aged 18-34 years exposed to physical violence during the past 12 months. The public health survey in Skåne 2008.

Figure 2 displays the setting of the exposure to physical violence. Some participants had experienced physical violence several times and in different locations, so the percentages add up to more than 100%. Most men (61%) had been violated in a public place (including streets, venues and transportation by bus, train or subway). Most women had been violated at home (37.7%) or in a public place (32%). Stratifying by age showed that younger women (18–24 years) were most often violated in public places, while somewhat older women (25–34 years) were most often violated at home. Among men there was no age difference regarding location of violence (data not shown).

## Discussion

The present study showed associations between experience of physical violence during the past year and self rated psychological health in women, but not men, aged 18–34 years. This is in accordance with results from some of the earlier population-based studies on psychological health and violence that included both men and women. A Danish study showed more than doubled odds of symptoms of anxiety and depression in women, but not men, who had been exposed to physical violence during the past year [22]. An Italian study of university students showed more than doubled odds of psychological distress in women, but not men, who had been exposed to a high degree of intimate partner violence (IPV) [23]. Some population studies have shown associations between violence and psychological distress in both men and women, but to a higher degree in women. A Swedish study of 17-year old students showed increased odds of psychological distress in girls and boys who had experienced physical violence during the past 12 months, OR: 2.68 for girls and OR: 1.90 for boys [2]. Another Swedish study on adults showed much higher odds of anxiety in women than men who had been exposed to physical violence or threats of violence during the past 12 months [11]. A Finnish study of university students showed that exposure to violence (life-time prevalence) was strongly associated with poor mental health in both men and women, with higher symptom levels in female victims [6]. Furthermore, population-based studies excluding men have shown significant associations between psychological distress and experience of violence in women [7,12-14,24].

In consistency with earlier research we found strong associations between poor self rated psychological health and socioeconomic status (especially not being part of the workforce) and psychosocial factors among both men and women [17]. Furthermore, men of non-European origin reported poor psychological health more often than men born in Sweden. A similar but not significant pattern was seen among women. Earlier studies have shown that mental ill-health is more common among foreign-born compared to native-born Swedes, mainly due to poorer socio-economic living conditions [33].

In our study alcohol risk consumption was associated with poorer self rated psychological health among women but not among men. Studies have shown bi-directional relationships between high alcohol consumption and both anxiety disorders [20] and depression [21] among men and women. The paralleled increases of alcohol consumption and poor mental health in young people over the last 20 years, with a poorer development among women in both respects [16], may point to a connection.

Those who are socially and/or economically disadvantaged are much more likely to experience violence [3,10]. This might in part be due to the fact that they are often restricted to live in neighborhoods with higher crime rates [34] and that problems with financial resources are linked to a range of negative outcomes including violence [3,35]. The fact that socioeconomic status is associated with both violence and psychological health has been taken into account by adjusting for socioeconomic status as a confounder in the analysis. Alcohol is a risk factor for violence [15,36], but neither a necessary nor sufficient cause [37,38]. It plays a larger part in situational violence than in controlling violence in intimate relationships [39]. Being violated may cause serious damage to basic trust. In our study a higher proportion of men and women who had been violated reported low trust compared to men and women who had not been exposed to violence.

Our results showed that men were most often violated in public places including streets, venues, buses, trains and subways. Women were most often violated at home, although younger women (18–24 years) were also most often violated in public places. This could be a reflection of different life styles in the two age groups. In a Swedish study, foreign-born women 18–64 years reported twice as much exposure to physical violence in the home compared to Swedish-born women [40].

The severity of symptoms may be influenced by the victim-offender relationship [2,6]. Violence against women often occurs in a private, isolated context including an intimate relationship to the perpetrator, while the perpetrator of men often is unknown. Physical abuse among women is often combined with sexual and/or emotional abuse, whereas physical abuse among men often occurs in isolation [5]. Women are less able to protect/defend themselves against perpetrators [41] and often have concerns on how to protect their children [42]. Furthermore, social and economic inequalities make it harder for women to leave an abusive partner [22]. A Swedish study showed that 22% of women aged 18–24 years had experienced some type of violence (physical, sexual or threats of violence) during the past year, and 85% worried about becoming victims of violence [38]. There might be some connection between the concurrent high prevalence of worrying about violence and the high prevalence of poor psychological health among young women in Sweden, as worrying is negatively related to psychological health [43].

The impact of violence on men’s health needs to be further explored. Abuse against men is highly prevalent in Sweden. A population-based study showed that 68% of Swedish men had experienced threats of violence and/or violence at some point during their lifetime and 14% during the past 12 months. The most common forms of violence were threatening or aggressive language and physical assaults, and many men had been victimized several times [4]. Victimization of men has been shown to be associated with health issues such as alcohol use problems [6,23,44]. It is possible that other measures than GHQ-12 might better capture psychological consequences of violence among men.

### Strengths and limitations

The current study is subject to some limitations. Firstly, the study is cross-sectional. A cross-sectional design makes it formally hard to infer causality, although such studies may well form at least part of causal inferences. Secondly, we had only one question on physical violence and one on location, but none on frequency or relationship to the perpetrator. Thirdly, in our study 9.7% of the men and 5.0% of the women 18–34 years reported experience of physical violence during the past year. This is in line with 12% of the men and 6% of the women 16–24 years reported in The Swedish National Public Health Surveys statistics 2006–2008 [45], which used the same single question on physical violence. However, this is probably an underestimation. Studies with several detailed questions on physical violence have reported considerably higher figures; 28% of the men and 11% of the women 16–24 years in a Danish national health interview survey [22], and 25% of the boys and 15% of the girls in a Swedish study of 17 year old high school students [2]. Earlier studies have also stated that violence against women is heavily underreported [1,8]. For example, the Swedish National Council for Crime Prevention has estimated that 75–80% of the cases of domestic violence go unreported [46].

We have explored the association between poor psychological health and violence, but of course there could be other factors contributing to emotional distress that we lack information on in this study (e.g., relationship problems, illness in the family, the demise of loved ones). The item we have used to measure generalized trust in other people is self rated and thus might be difficult to validate, but it has been used in many previous investigations [32]. Strengths of this study are the large population sample, the use of the well-validated GHQ-12 measure to assess psychological health and the use of a questionnaire to assess exposure to violence [47]. Although there are more complex GHQ-12 instruments (with for example 28 and 60 items) to measure psychological health, there is little difference in validity [26,28]. Furthermore, the GHQ-12 measure, as well as the question used to assess experience of physical violence, has been validated by the National Institute of Public Health and by Statistics Sweden [29].

## Conclusions

In this study women, but not men, aged 18–34 years, who had experienced physical violence during the past year showed more than doubled odds of poor psychological health. The association between experienced physical violence and poor psychological health found in women persisted, although attenuated, after adjustment for covariates. There was a gender difference regarding location of violence, as men were mostly violated in public places while women were most often violated at home. It is well known that domestic violence has serious consequences on physical and mental health, both in a short and long perspective. Mental ill-health and violence are both important public health issues and the impact of violence on mental health should be further explored among both genders. Awareness of gender differences regarding context and mental impact of violence may assist public health workers in reducing the consequences of violence and to design preventive strategies.

## Competing interests

The authors declare that they have no competing interests.

## Authors’ contributions

MF, ML and MR have contributed to the conception and drafting of the work. MF has analyzed the data and written the first draft of the manuscript. MF, ML and MR have contributed to the interpretation and the discussion of the results, and the revision of the content. All authors have read and approved the final manuscript.

## Pre-publication history

The pre-publication history for this paper can be accessed here:

http://www.biomedcentral.com/1471-2458/14/29/prepub
